# Effects of precipitation and temperature on precipitation use efficiency of alpine grassland in Northern Tibet, China

**DOI:** 10.1038/s41598-020-77208-6

**Published:** 2020-11-20

**Authors:** Xiaoke Zhang, Xindong Du, Zhangming Zhu

**Affiliations:** 1 School of Public Administration, Hohai University, Nanjing, 210098 Jiangsu China; 2 School of Ecology and Environmental Science and Yunnan Key Laboratory for Plateau Mountain Ecology and Restoration of Degraded Environments, Yunnan University, Kunming, 650091 China

**Keywords:** Grassland ecology, Ecological modelling

## Abstract

Precipitation use efficiency (PUE) is crucial in understanding the coupling between ecosystem carbon and water cycling. In this study, we used a time series (2000–2013) dataset of net primary productivity (NPP) based on the Carnegie–Ames–Stanford Approach (CASA) model together with precipitation to reveal the spatial and temporal patterns of alpine grassland PUE in Northern Tibet. The mean annual PUE values of alpine meadow, alpine meadow steppe, alpine steppe, alpine desert steppe, and alpine desert were 0.48, 0.39, 0.36, 0.29 and 0.23 gc m^−2^ mm^−1^, respectively. The spatial patterns of PUE of alpine grassland demonstrated an initial increase in the arid region and a subsequent decrease in the humid region along the precipitation gradient and peaked at approximately 500 mm. To evaluate the temporal patterns, the sensitivity $Slope$ and the Pearson correlation coefficient ${R}_{xy}$ between the PUE and climatic factors were calculated. The inter-annual variability of PUE exhibited a significant negative correlation with annual precipitation (P < 0.05), which implies that NPP had a lower sensitivity to precipitation in most regions. The relationship between PUE and the mean annual temperature is different for different regions. Our findings have an important role in understanding the impacts of precipitation availability on climate change and in the scientific management of the alpine grassland ecosystems.

Net primary productivity (NPP) is an important variable of terrestrial ecosystems, as it is an important indicator of the global carbon cycle^1,2^. Precipitation use efficiency (PUE), which is the ratio of NPP to precipitation, has been considered as an integral method for assessing the response of NPP to spatial and temporal variations of annual precipitation^3,4^. PUE is a considerable constraint for simulating ecosystem productivity in models^5^ and can be used as an indicator of regional degradation^6,7^. It is also essential in understanding the coupling between ecosystem carbon and water cycling^8^.

Recently, the PUE trends of grassland along the precipitation gradient have demonstrated various perceptions. In general, PUE decreased spatially with potential evapotranspiration and increasing aridity^4,9^. However, some studies found that PUE exhibited a unimodal pattern with an increasing trend in dry regions and a decreasing trend in mesic regions^10–12^. Huxman et al.^13^ found that PUE decreased with increasing precipitation and that there is a convergence to a common maximum PUE during the driest years at each of the sites they selected in America. In contrast, Hu et al.^14^ found that the maximum PUE exhibited large site-to-site variation along a 4500-km grassland transect. Thus, it is critical to reveal the patterns of PUE across the precipitation gradient in alpine grasslands in Northern Tibet.

Previous studies have suggested that PUE may be influenced by factors, such as edaphic conditions (soil texture and soil carbon content), vegetation conditions (vegetation cover and species richness), and biogeochemical constraints (soil nitrogen (N))^9,11,13,14^. Climatic factors also have a significant effect on PUE through influencing the ecosystem carbon cycle^3^. The PUE of grassland ecosystems has been reported to exhibit decreasing^9^, unchanging^15^ and increasing^16^ trends with increasing precipitation. In a mixed-grass prairie in USA, double precipitation suppressed the PUE and half precipitation increased the PUE under different treatments^17^. PUE either decreases^18^, exhibits no changes^17^, or increases^1^ under warming. For drought, Zhang et al.^3^ found that a wet year preceded by a dry year resulted in the lowest PUE and a dry year preceded by a wet year resulted in the highest PUE across China. From the point of view of ecosystem resilience, Campos et al.^19^ found higher PUE in drier years, which increased significantly with drought. Therefore, it is essential to have a deep understanding of how PUE responds to climate change to accurately forecast the ecosystem carbon cycle.

Many of the previous studies are based on the spatial patterns of PUE in the plot scale^9,11,15,17^. Little research has been conducted to study the PUE dynamics of the alpine grassland ecosystem in a regional scale and its driving forces. In this study, we investigated the spatial–temporal pattern and variation of PUE of alpine grasslands in Northern Tibet, over the time period from 2000 to 2013. Our goals were to: (1) determine the changes in the PUE pattern along the precipitation gradient and (2) explore the temporal relationship between PUE and climatic factors.

## Results

### Spatial patterns of PUE in Northern Tibet

**Figure 1:**
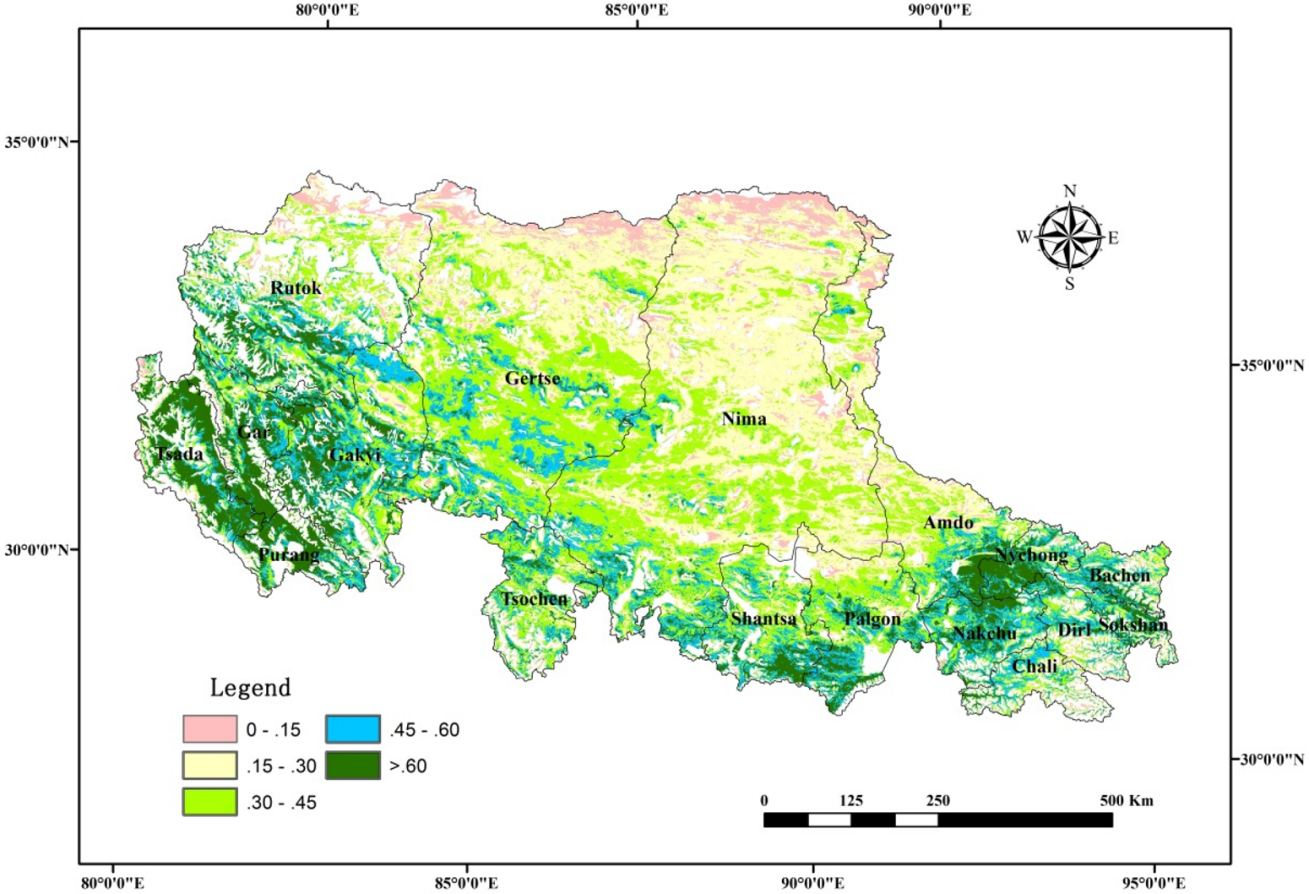
Spatial distribution of the mean annual PUE in Northern Tibet during 2000–2013. The map was created using ArcMap 10.6, URL: https://www.esri.com/en-us/arcgis/products/arcgis-pro/overview.

During 2000 and 2013, the spatial distribution of the mean annual PUE of the alpine grassland in Northern Tibet gradually decreased from south to north, which changed from more than 0.6 gc m^−2^ mm^−1^ in the south and descended to less than 0.15 gc m^−2^ mm^−1^ in the north (Fig. 1). For different alpine grassland classes, the mean annual PUE values were in the order of alpine meadow > alpine meadow steppe > alpine steppe > alpine desert steppe > alpine desert. The PUE values were 0.48, 0.39, 0.36, 0.29 and 0.23 gc m^−2^ mm^−1^, respectively (Table 1).

**Table 1:** MAP, NPP, and PUE of different alpine grassland types in Northern Tibet during 2000–2013.

Alpine grassland type	Area/km^2^	MAP/(mm a^−1^)	NPP/(gc m^−2^ a^−1^)	PUE/(gc sm^−2^ mm^−1^)
Alpine meadow	99,056	501.26	238.94	0.48
Alpine meadow steppe	52,618	365.99	143.89	0.39
Alpine steppe	256,331	246.61	88.06	0.36
Alpine desert steppe	99,056	181.77	53.18	0.29
Alpine desert	50,451	189.27	43.29	0.23

MAP, multi-year mean annual precipitation.

### PUE variation along the precipitation gradient

**Figure 2:**
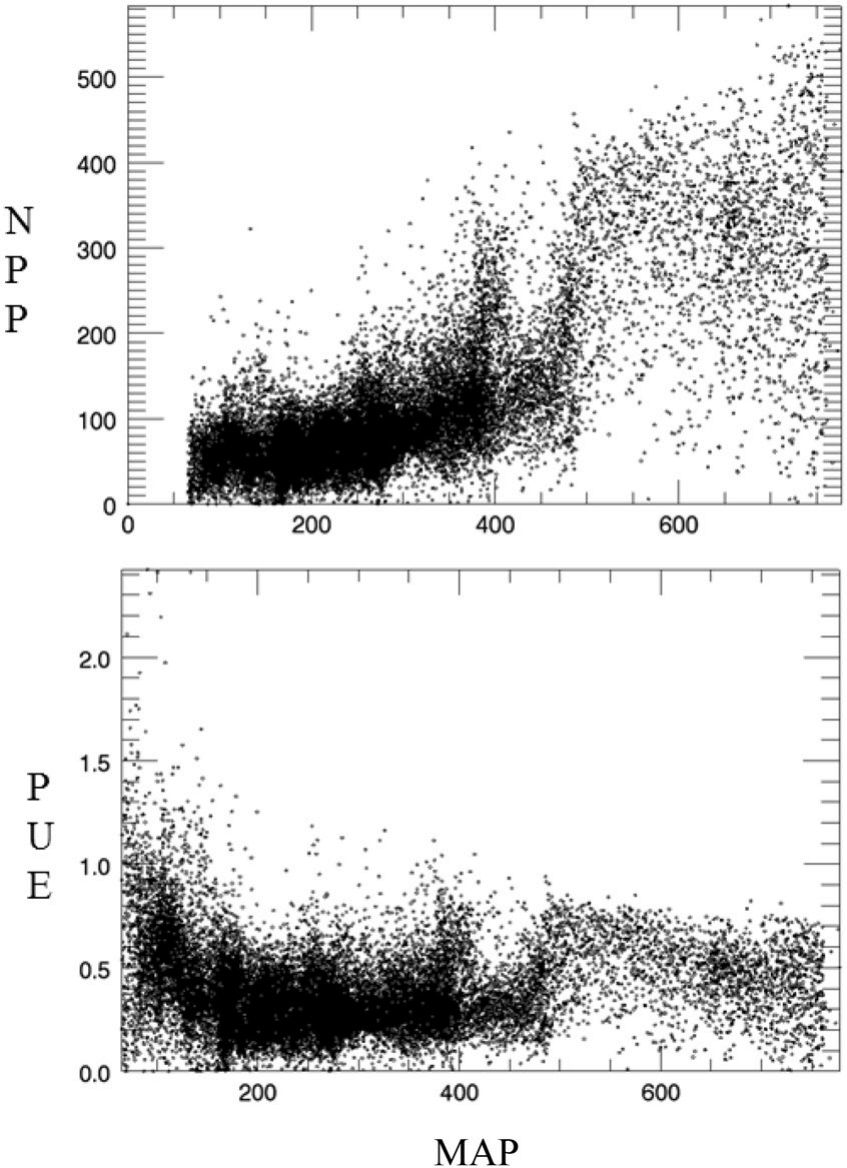
NPP and PUE patterns along the precipitation gradient.

Figure 2 illustrates that NPP in the alpine grassland was positively correlated with the multi-year mean annual precipitation (MAP). While MAP is more than 400 mm a^−1^ in the alpine meadow, change in the NPP trend is not obvious along the precipitation gradient. The PUE of the alpine grassland exhibited an initial increase and a subsequent decrease along the precipitation gradient. The PUE increased with MAP in the drier portions of the precipitation gradient. In contrast, the PUE decreased with MAP in the wetter portions of the precipitation gradient. Thus, PUE of the alpine grasslands peaked at approximately 500 mm of MAP.

### Relationship between PUE and Climatic Factors

**Figure 3:**
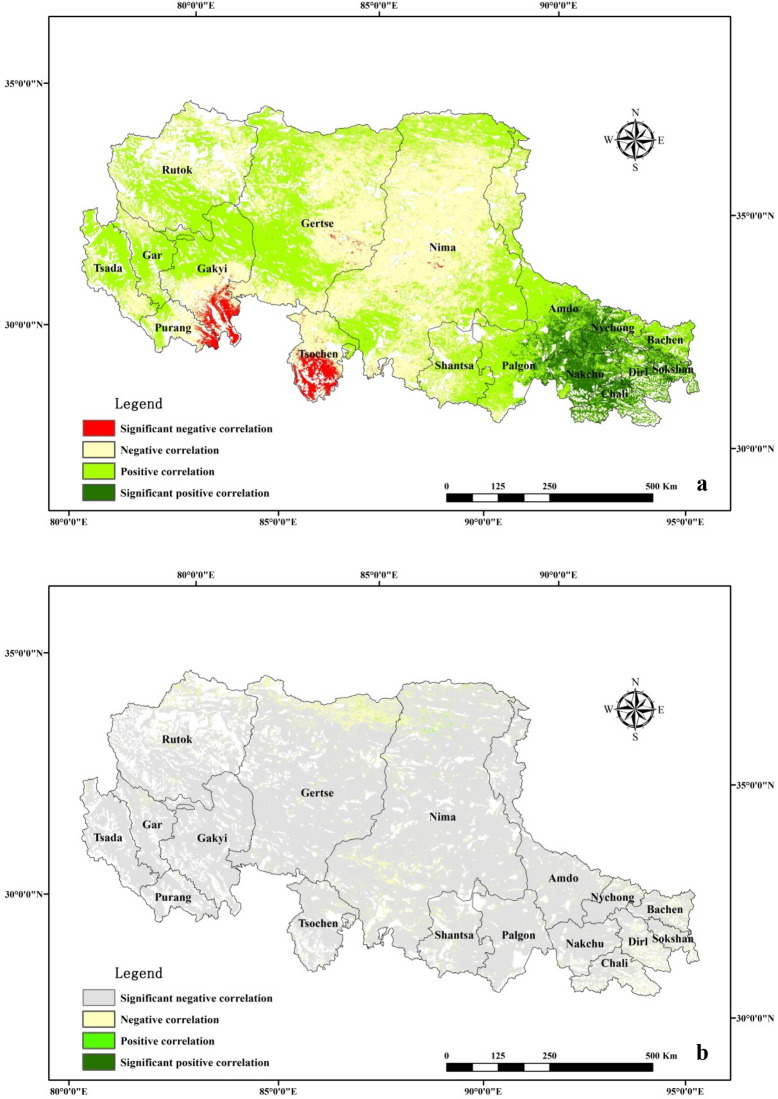
Correlation between PUE and (**a**) temperature, (**b**) annual precipitation during 2000–2013. The map was created using ArcMap 10.6, URL: https://www.esri.com/en-us/arcgis/products/arcgis-pro/overview.

The correlation coefficients between PUE and climatic variables differed for different alpine grasslands (Fig. 3). The inter-annual variability of PUE exhibited a significant negative correlation with annual precipitation (P < 0.05).

Nevertheless, the relationship between PUE and the mean annual temperature is different for different regions. In the southeast-distributed alpine meadow, PUE exhibits a significant positive correlation with the mean annual temperature (P < 0.05). In the south of Gakyi and Tsochen counties, PUE exhibits a significant negative correlation with the mean annual temperature (P < 0.05). In the middle of the study region, PUE exhibits a negative correlation with the mean annual temperature and a positive correlation with the mean annual temperature in other regions.

## Discussion

### PUE pattern in Northern Tibet

For different alpine grassland classes, the mean annual PUE values exhibited greater differences. Alpine meadow exhibited a maximum PUE value of 0.48 gc m^−2^ mm^−1^, whereas the alpine desert exhibited a minimum PUE value of 0.23 gc m^−2^ mm^−1^. The spatial pattern of the mean annual PUE for different alpine grassland types was congruent with those of previous studies^8,11,20^. The drier the climate, the higher the water consumption coefficient and the lower the PUE. The reason for this may be largely due to their differences in vegetation cover, species richness, soil texture, soil carbon content and soil N^9,11,14^.

Our results also supported the view that PUE exhibited a unimodal pattern across the precipitation gradient, with an increasing trend in the dry regions and a decreasing trend in the humid regions, which was similar to that in global grasslands^10,11^. Some studies were inconsistent with the findings of this study. Lauenroth et al.^15^ observed that the PUE of native grasslands exhibited no significant change across the precipitation gradient in the United States. Hu et al.^14^ and Bai et al.^9^ found that PUE increased across the precipitation gradient. Therefore, the PUE patterns at different scales were not the same.

For the PUE peak value, Paruelo et al.^10^ found that PUE peaked at approximately 475 mm. Hu et al.^14^ considered that the maximum value point may be in a MAP range of 400–600 mm, which is quite close to this study. In the humid region, the lower PUE may have been caused by higher runoff, or higher evapotranspiration rate^14,21^. In the arid region, the lower PUE could be caused by lower NPP, higher evaporation, or higher water limitation^9,19^. Therefore, effective precipitation is the main factor that can control the PUE patterns.

### Impact of climatic factors on PUE

Figure 3 depicts the relationship between the PUE and temperature in this study. The influence of temperature on PUE is extremely complicated and distinct for the different regions. While the temperature changes, the change in PUE may be through stomatal control mechanisms^9^. The optimum temperature of plant photosynthesis demonstrates a large variability for different environmental conditions. When the environment temperature change is below the optimum temperature of photosynthesis, the influence of temperature on PUE is positive, or vice versa^22,37^. In the future, warming in humid regions may continue to have positive effects on PUE; warming in arid regions induced by drought may strengthen its negative effects on PUE of alpine grasslands.

**Figure 4:**
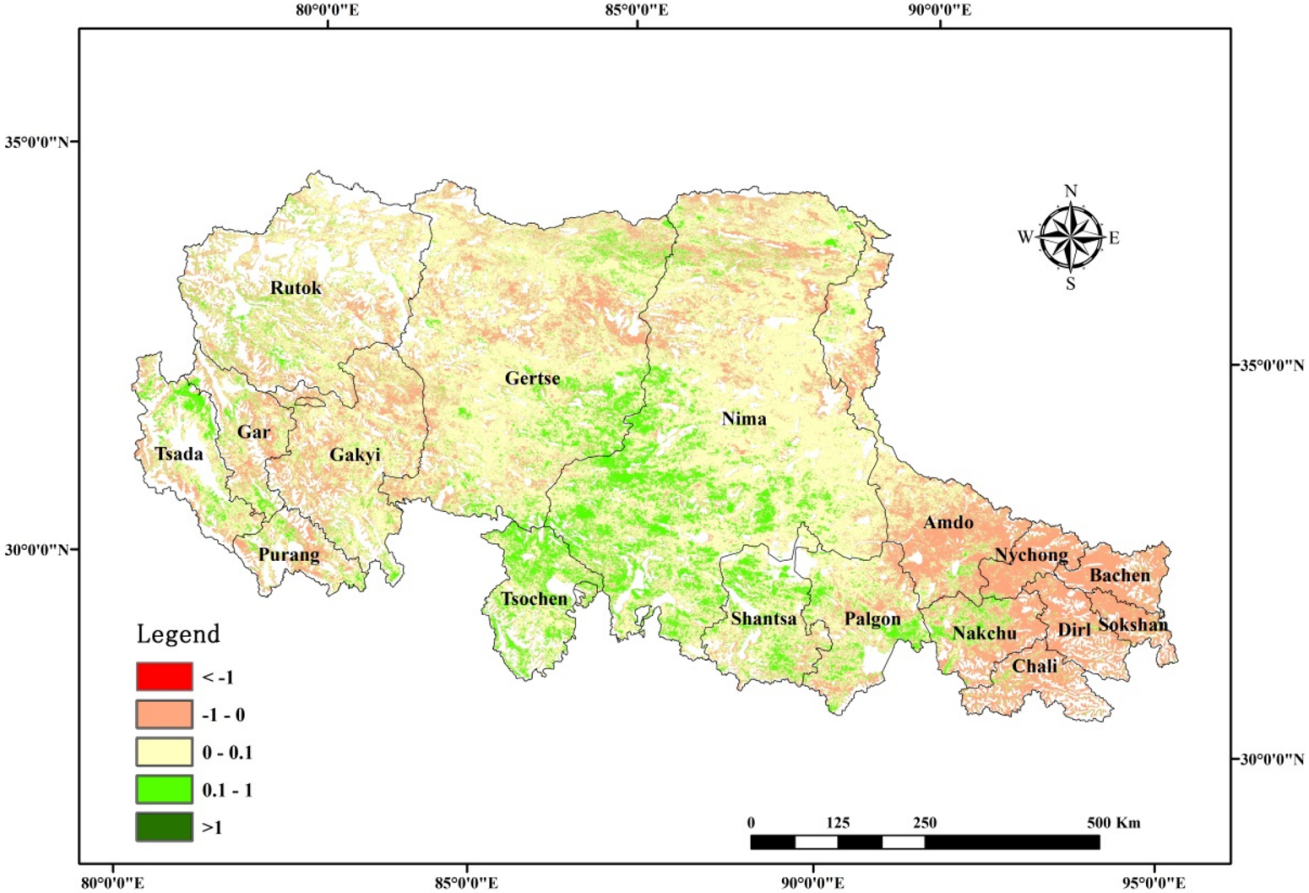
Sensitivity $Slope$ between NPP and precipitation. The map was created using ArcMap 10.6, URL: https://www.esri.com/en-us/arcgis/products/arcgis-pro/overview.

In this study, the temporal correlation between PUE and annual precipitation was negative, which was consistent with the findings of Chen et al.^9^. Figure 4 depicts that NPP exhibits a lower sensitivity to precipitation; the slope value between NPP and precipitation is between − 1 and 1 in most regions. NPP increases with precipitation, as the slope value is greater than 0 but less than 1 in the central regions. This illustrates that when the precipitation increases by 1 mm, NPP increases by less than 1 gc m^−2^ a^−1^. The inter-annual variation of precipitation is larger than that of the NPP variation, i.e., the annual precipitation increases, NPP increases; as the NPP increase in amplitude is smaller, the PUE (NPP/MAP) decreases. NPP decreases with increasing annual precipitation in the meadow and in some other sporadic regions. As the annual precipitation increases, NPP decreases and PUE (NPP/MAP) also decreases. Therefore, the inter-annual variability of PUE exhibited a significant negative correlation with the annual precipitation. For further studies, we need to clarify the internal mechanisms influencing the PUE variations at different scales.

## Methods

### Study area

**Figure 5:**
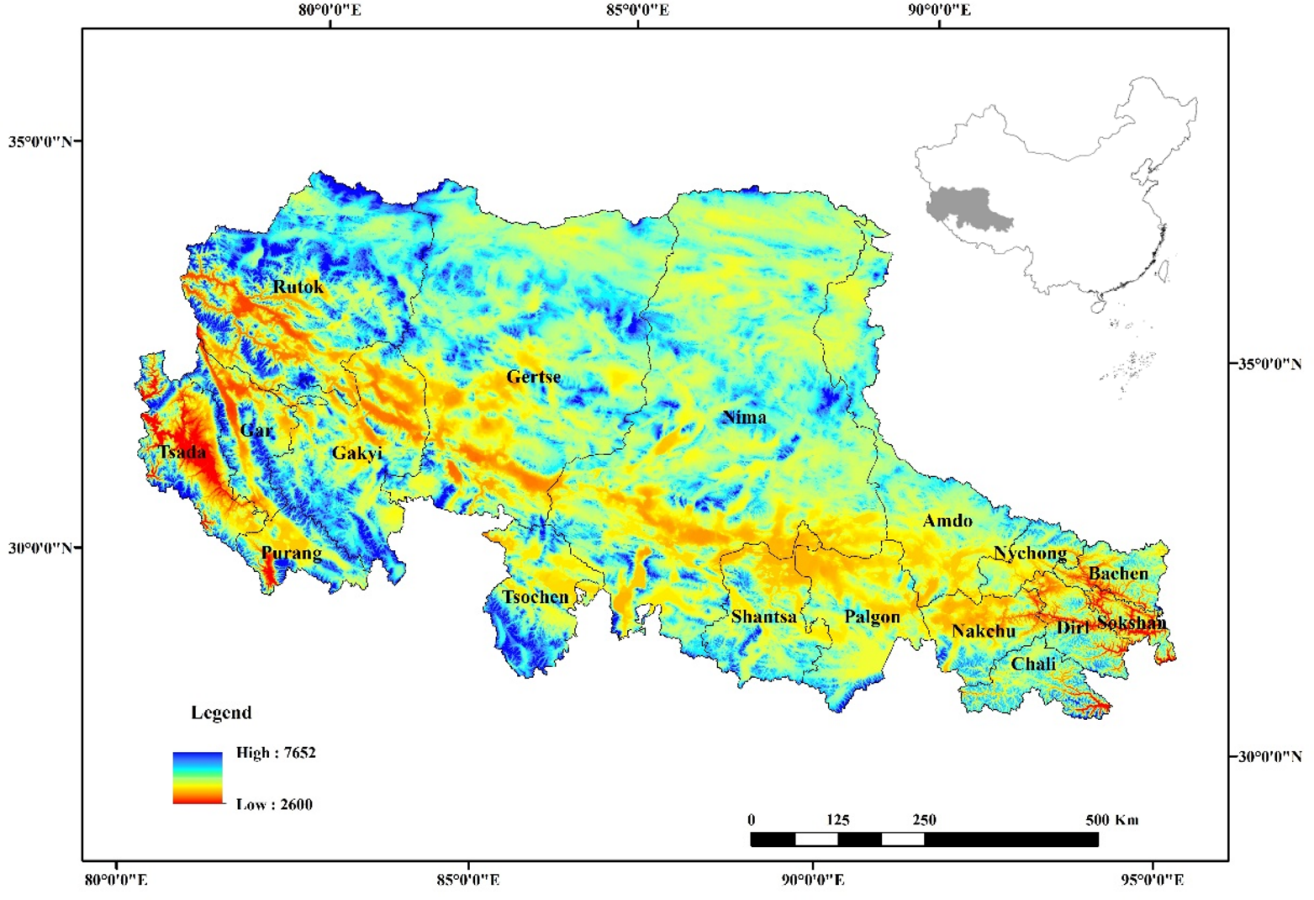
Location and topography in Northern Tibet, China. The map was created using ArcMap 10.6, URL: https://www.esri.com/en-us/arcgis/products/arcgis-pro/overview.

As the sole and largest geographical part at the highest elevation on earth, the Qinghai–Tibet plateau is called the “Third Pole”. It acts as an important reservoir for water and regulates the water resources and climatic conditions of East Asia and as well as those of the whole world^23,24^. The study area, Northern Tibet, is an important part of the Qinghai-Tibet plateau and includes the Ngari and Nakchu prefectures with an average altitude of more than 4000 m (Fig. 5). The climate of the entire area is extremely cold and dry, and hydrothermal conditions are very harsh. The mean annual temperature ranges from approximately − 1.8 °C to 4.2 °C. The annual precipitation varies between 67.5 and 752.3 mm and is less than 100 mm in Gar county. The annual precipitation in this region declines from south to north and from east to west, and is mostly concentrated from May to September, a period that accounts for 90% of the annual precipitation. High altitude western winds are strong in spring and winter, and gales above force 7 are frequent and occur for over 100 days annually, which leads to dry weather and low soil temperature^25,26^. Alpine grassland, mainly comprised of alpine meadows and steppes, is the main vegetation type across much of the region, covering 94.4% of the total area in Northern Tibet, which is sensitive to the variable environment of Northern Tibet^27^.

### Data

In this study, we used the MODIS NDVI time series of satellite images at 250 km spatial and 16-day temporal resolution, covering a period from 2000 to 2013. The product MOD13Q1 was obtained from the website https://ladsweb.nascom.nasa.gov/data/search.html. We acquired five MODIS tiles (h24v05, h25v05, h25v06, h26v05 and h26v06) for our study region. The MODIS tiles were mosaicked and re-projected from the Sinusoidal projection to the Albers projection. Monthly NDVI images were generated by the maximum NDVI value composite (MVC) method using the two 16-day composites for each month^28,29^.

**Figure 6:**
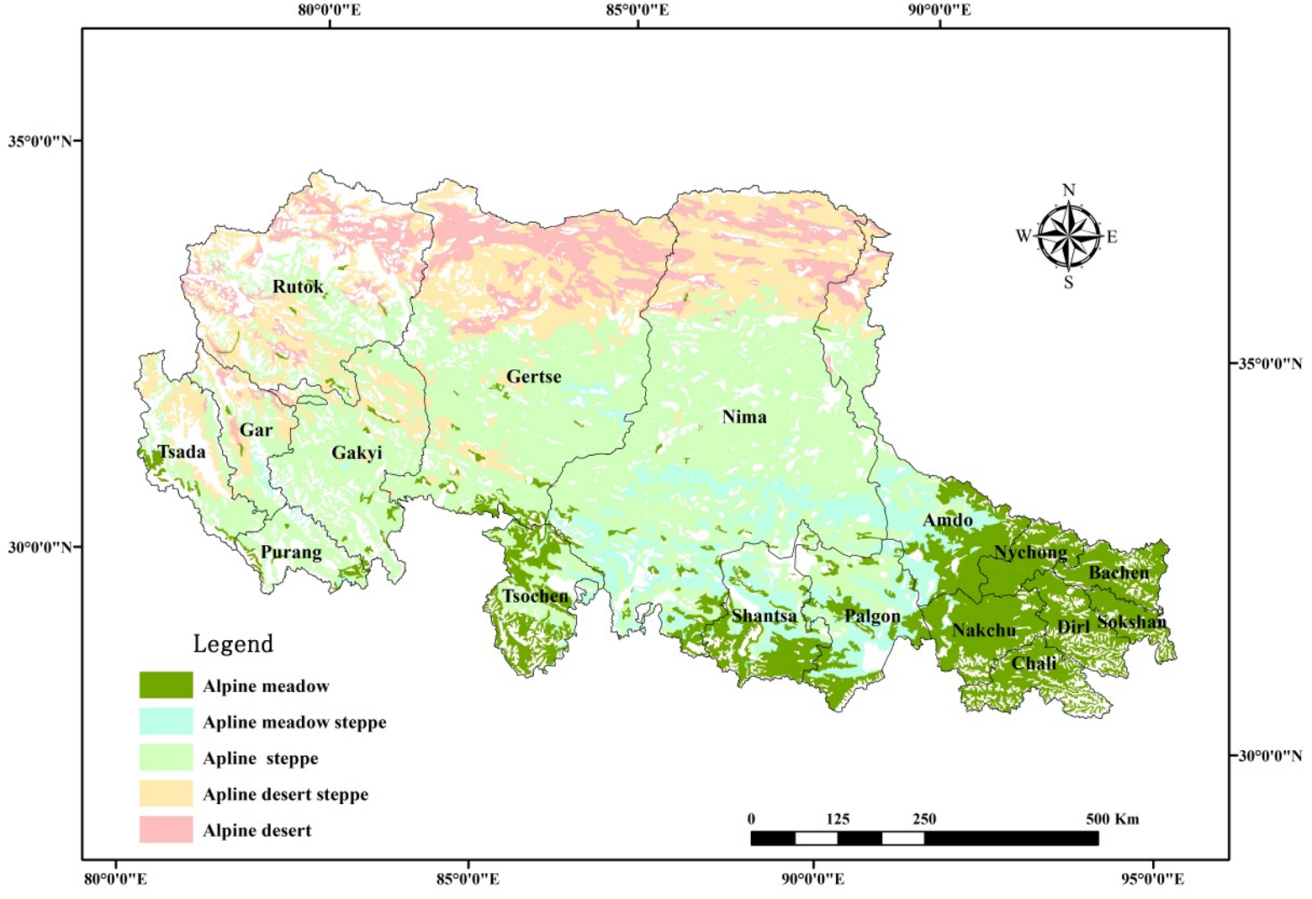
Spatial distributions of alpine grassland classes in Northern Tibet. The map was created using ArcMap 10.6, URL: https://www.esri.com/en-us/arcgis/products/arcgis-pro/overview.

The mean annual temperature and annual precipitation were generated by datasets (SURF_CLI_CHN_PRE_MON_GRID_0.5 and SURF_CLI_CHN_TEM_MON_GRID_0.5), which were derived from the China Meteorological Data Sharing Service System (https://data.cma.cn). To match a spatial resolution of 250 m × 250 m, datasets were produced by ordinary kriging spatial interpolation^29^. Grassland classification data were obtained from the grassland resources of Tibet autonomous region. Alpine grasslands were identified from the southeast to northwest: alpine meadow, alpine meadow steppe, alpine steppe, alpine desert steppe, and alpine desert (Fig. 6)^29^.

### Methods

The Carnegie–Ames–Stanford Approach (CASA) model was developed to calculate NPP on a large scale, based on NDVI, climate, and classification data. It was determined by two parts: absorbed photosynthetically active radiation ($APAR$) and light use efficiency ($\varepsilon$). The formula is as follows:

$$NPP\left(x,t\right)=APAR\left(x,t\right)*\varepsilon \left(x,t\right)=IPAR\left(x,t\right)*FPAR\left(x,t\right)*{\varepsilon }_{max}*{T}_{\varepsilon 1}\left(x,t\right)*{T}_{\varepsilon 2}\left(x,t\right)*{W}_{\varepsilon }\left(x,t\right)$$

$$IPAR\left(x,t\right)=SOL(x,t)*0.5$$

$$FPAR\left(x,t\right)=\frac{(NDVI(x,t)-{NDVI}_{i,min})\times ({FPAR}_{max}-{FPAR}_{min})}{({NDVI}_{i,max}-{NDVI}_{i,min})}+{FPAR}_{min}$$

$$FPAR\left(x,t\right)=\frac{(SR(x,t)-{SR}_{i,min})\times ({FPAR}_{max}-{FPAR}_{min})}{({SR}_{i,max}-{SR}_{i,min})}+{FPAR}_{min}$$

$$SR\left(x,t\right)=\left[\frac{1+NDVI(x,t)}{1-NDVI(x,t)}\right]$$

$$FPAR\left(x,t\right)={\alpha FPAR}_{NDVI}+(1-\alpha ){FPAR}_{SR}$$

$${T}_{\varepsilon 1}=0.8+0.02\times {T}_{opt}\left(\mathrm{x}\right)-0.0005\times {[{T}_{opt}(\mathrm{x})]}^{2}$$

$${T}_{\varepsilon 2}\left(x,t\right)=\frac{1.184}{\left\{1+\mathrm{exp}\left[0.2\times \left({T}_{opt}\left(x\right)-10-T\left(x,t\right)\right)\right]\right\}}\times \frac{1}{\{1+\mathrm{exp}[0.3\times (-{T}_{opt}\left(x\right)-10+T(x,t))]\}}$$

$${W}_{\varepsilon }\left(x,t\right)=0.5+0.5\times E(\mathrm{x},\mathrm{t})/{E}_{p}(x,t)$$

$$E\left(x,t\right)=\left\{P\left(x,t\right)\times {R}_{n}\left(x,t\right)\times \left[{\left(P\left(x,t\right)\right)}^{2}+{\left({R}_{n}\left(x,t\right)\right)}^{2}+P\left(x,t\right)+{R}_{n}\left(x,t\right)\right]\right\}$$

$$/\{[P\left(x,t\right)+{R}_{n}\left(x,t\right)]\times [{\left(P\left(x,t\right)\right)}^{2}+{\left({R}_{n}\left(x,t\right)\right)}^{2}]\}$$

$${R}_{n}\left(x,t\right)={[{E}_{p0}(x,t)\times P(x,t)]}^{0.5}\times \{0.369+0.598\times {[\frac{{E}_{p0}(x,t)}{P(x,t)}]}^{0.5}\}$$

$${E}_{p}\left(\mathrm{x},\mathrm{t}\right)=\left[E\left(x,t\right)+{E}_{p0}(x,t)\right]/2$$

Here, $IPAR$ is the interrupted photosynthetically active radiation; $SOL(x,t)$ is the total solar radiation based on sunshine duration; $FPAR$ is fraction of the photosynthetically active radiation absorbed by the canopy, which is calculated from the MODIS NDVI; ${\varepsilon }_{max}$ is the maximum light use efficiency as 0.608 gC M J^−1^^26^; ${T}_{\varepsilon }and {W}_{\varepsilon }$ are the unitless stress values for temperature and water; ${T}_{opt}\left(x\right)$ is the air temperature (°C) in the month when NDVI reaches its maximum for the year; $E(\mathrm{x},\mathrm{t})$ is the estimated evapotranspiration (mm); ${E}_{p}(x,t)$ is the potential evapotranspiration (mm); $P\left(x,t\right)$ is the precipitation (mm); ${R}_{n}\left(x,t\right)$ is the net solar radiation (MJ) and ${E}_{p0}(x,t)$ is the local potential evapotranspiration (mm); respectively^24,30–32^.

To analyse the temporal variation of the alpine grassland PUE in Northern Tibet, the sensitivity $Slope$^21^ and the Pearson correlation coefficient ${R}_{xy}$^33,34^ between the PUE and climatic factors were calculated.

$$Slope=\frac{\sum_{i=1}^{n}({x}_{i}-\overline{x})(y-\overline{y})}{\sum_{i=1}^{n}{(x-\overline{x})}^{2}}$$

$${R}_{xy}=\frac{\sum_{i=1}^{n}({x}_{i}-\overline{x})(y-\overline{y})}{\sqrt{\sum_{i=1}^{n}{{({x}_{i}-\overline{x})}^{2}\sum_{i=1}^{n}(y-\overline{y})}^{2}}}$$

Here, $\overline{x}$ and $\overline{y}$ are respectively the multi-year mean of the climatic factors and the PUE; ${x}_{i}$ and ${y}_{i}$ are respectively the climatic factors and PUE of the year $i$. If ${R}_{xy}$ passes through the significance test (P < 0.05), it exhibits a significant positive or negative correlation.

### Validation of NPP

**Figure 7:**
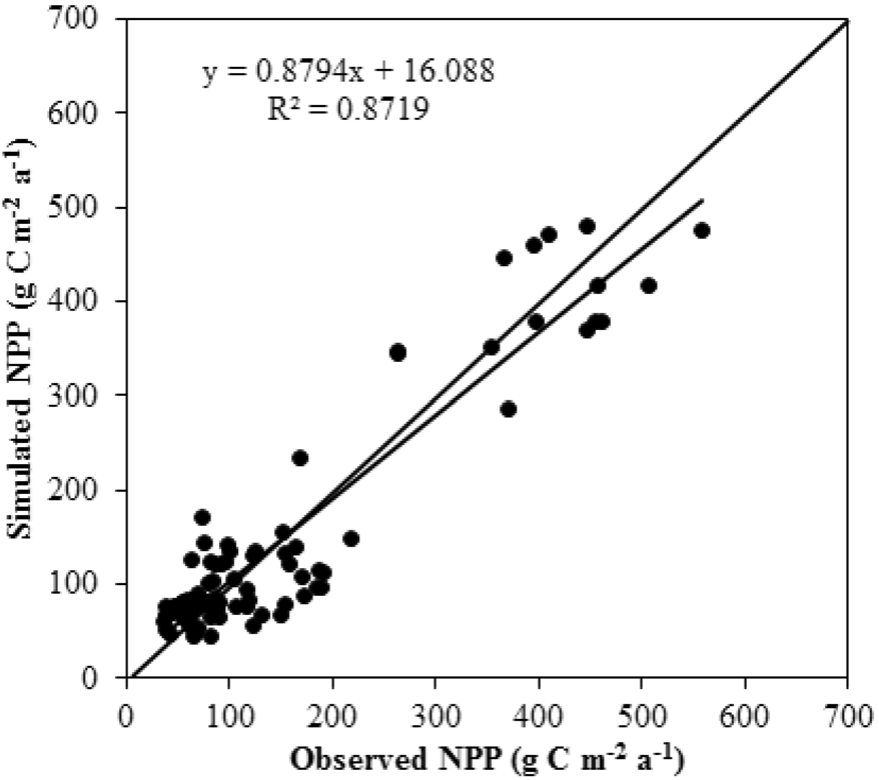
Comparison of observational data with simulation results.

To validate the NPP simulated by the CASA model, biomass was measured in the field^39^. The alpine grassland NPP for both aboveground (ANPP) and belowground (BNPP) was calculated using the same method as that proposed by Chen et al.^35^ A significant linear relationship was observed between the observed and simulated NPP (P < 0.01) (Fig. 7). We compared our results with the results from other studies (Table 2), and found that the NPP calculated by the CASA model was reliable.

**Table 2:** Mean annual NPP simulated for alpine grasslands by different researchers (gc m^−2^ a^−1^).

Grassland type	This study	Chen et al.^35^	Piao et al.^36^	Gao et al.^37^	Zhou et al.^38^
	CASA	GLO-PEM	CASA	CASA	TEM
Alpine meadow	239	301	176	63.5	214.6
Alpine steppe	86	70	80	30.6	64
Alpine desert	43	18	24	15.6	

## Data Availability

The datasets generated and/or analyzed during the current study are available from the corresponding author on reasonable request.
